# Futures literacy as a research tool for socio-material revaluation in sufficiency-oriented climate mitigation

**DOI:** 10.3389/fsoc.2026.1824778

**Published:** 2026-09-25

**Authors:** Ioannis Theocharis, Björn Bohnenkamp

**Affiliations:** Karlshochschule International University, Karlsruhe, Germany

**Keywords:** anticipatory assumptions, circular consumption, demand-side mitigation, futures literacy, just transitions, participatory methods, socio-material revaluation, sufficiency

## Abstract

Demand-side climate mitigation requires methodological approaches capable of interrogating how everyday material practices are shaped by anticipatory assumptions about the future. While decluttering has been discussed as a potential entry point for sufficiency-oriented consumption, existing approaches largely focus on individual reflection and functional evaluation, leaving collective and anticipatory dimensions underexplored. This article develops and systematizes an adapted Futures Literacy Lab (FLL) as a methodological framework for examining socio-material revaluation processes in the context of stored household consumer goods. Building on Futures Literacy theory, the method operationalizes revaluation through structured phases—Reveal, Reframe, and Rethink—supplemented by an optional Act phase. Through developmantalworkshops, three ideal-typical workshop formats were derived: contextual thematic futures, object-centered future projection, and scenario-based reframing. Rather than presuming immediate behavioral change, the framework makes anticipatory assumptions visible and negotiable through collective intelligence knowledge creation, thereby expanding interpretative space relevant to sufficiency transitions. The article contributes a transferable methodological approach for investigating and facilitating socio-material revaluation in high-consumption contexts, positioning anticipatory imagination as a complementary tool within climate mitigation and just transition research.

## 1 Introduction

Since the 1990s, global energy and material consumption have risen far beyond the levels required to secure decent living standards (Rao and Min, 2017; Millward-Hopkins et al., 2025). This expansion is particularly pronounced in high-income countries of the Global North, where structural energy surplus and high material throughput are decoupled from basic needs satisfaction. According to the United Nations Environment Programme (2024), extraction and processing account for approximately 55% of global greenhouse gas emissions and 90% of land-use–related biodiversity loss. Simultaneously, the total mass of anthropogenic materials now exceeds that of all living biomass (Elhacham et al., 2020). A significant share of these materials is embedded in consumer goods that remain unused yet retained in private households (Wieser et al., 2023), often stockpiled in attics, basements, and garages—spaces described as “dead ends” within linear consumption systems (Eimterbäumer and Jaeger-Erben, 2025).

From a climate mitigation perspective, these dormant household stocks represent both accumulated past emissions and unrealized sufficiency potential. If reactivated and recirculated—through reuse, sharing, repurposing, or redistribution, they could reduce aggregate demand for newly produced goods and thus contribute to demand-side mitigation strategies aligned with high-impact circular economy measures such as Reduce, Refuse, and Reuse. This demand-side potential carries a distinct climate justice dimension. Millward-Hopkins et al. (2025) demonstrate that growth in energy and material consumption is occurring primarily in high-income countries that already exceed sufficiency thresholds, while many lower-income countries still fall short of the levels required to secure decent living standards for their populations. Reducing surplus material throughput in affluent Global North households is therefore not only a mitigation measure but a question of distributive justice: the environmental burdens of extraction and production that sustain overconsumption in materially affluent contexts are disproportionately borne by communities elsewhere and by future generations. Addressing the anticipatory assumptions that normalize high material throughput thus forms part of a broader just transition agenda, linking household-level revaluation to structural questions of global equity. However, empirical studies indicate that stored goods rarely re-enter circulation automatically. Their activation depends on processes of revaluation that transform objects from privately reserved possessions into relational resources embedded in broader socio-material networks (Hipp and Jaeger-Erben, 2023). Decluttering has therefore emerged as a potential entry point for sufficiency-oriented consumption change (Muster et al., 2022).

Existing research on reflective decluttering demonstrates that structured reflection during sorting processes can reduce short-term acquisition and stimulate learning about consumption habits (Iran et al., 2024; Muster et al., 2022). Yet these approaches conceptualize decluttering primarily as a functional and present-oriented evaluative process, focusing on utility, emotional attachment, or lifestyle fit. Reflection is typically deployed to optimize current consumption patterns rather than to challenge the anticipatory assumptions that underpin them. As elaborated further in Section 2.2 decluttering thus remains framed as an individual sorting activity rather than as a collective act of socio-material future-making.

What remains methodologically unexamined is whether anticipatory imagination—specifically, the speculative reconfiguration of possible futures for objects, practices, and identities—can function as a transformative enabler toward sufficiency. Existing decluttering methods do not systematically engage participants in structured processes that reveal and reconfigure the anticipatory assumptions guiding decisions about retention, disposal, and acquisition. Nor do they conceptualize decluttering as a collective intelligence knowledge creation process capable of making these assumptions explicit and analyzable. As a result, the mechanisms through which perspective shifts might destabilize consumption norms and reconfigure household material stocks in climate-relevant ways remain methodologically underdeveloped.

To address this gap, this paper develops a conceptual—methodological adaptation framework of UNESCO (2026) the Futures Literacy Lab (FLL) tailored to household decluttering contexts. A Futures Literacy Lab is a participatory workshop format, developed by UNESCO (2026) that helps people explore how they imagine the future and use those assumptions to rethink the present. It is less about predicting what will happen and more about revealing, reframing, and questioning the stories and expectations people already carry about the future. The capability that is cultivated during an FLL workshop is called Futures Literacy. A Futures Literate person has the capability to identify, make explicit and adapt the ways people or institutions use ideas of the future. These ideas of the future are according to Miller anticipatory systems (Miller, 2018; p.2). Building on the standard FLL design and on developmental workshops, the adapted version uses this capability of consciously using anticipatory systems on decluttering practices to explore the anticipatory imagination behind storing objects and its potential effects on decluttering practices. Rather than focusing solely on functional evaluation of possessions, the laboratory format invites participants to collaboratively explore alternative futures for their belongings, identities, and practices.

As shown in section 2.1 the decluttering practices examined are individualistic. By embedding decluttering within FLL design, the practice is shifted from an individual sorting activity toward a collective negogiated one. Escaping individualistic tendency in decluttering practicing is happening through the central part in the FLL Design which according to (Miller 2018, p.107) is the Collective Intelligence Knowledge Creation (CIKC) process. This is conceptualized by (Miller 2018, p.107) as a generative process through which participants—situated in specific cultural and temporal contexts—collaboratively shift tacit anticipatory assumptions into explicit, negotiable knowledge, thereby creating conditions for the emergence of genuinely novel collective understanding.

The framework is deliberately designed to operate at the collective rather than the individual level, engaging the well-established critique that household-level sufficiency interventions risk individualizing structural responsibility for climate change by locating the causes of overconsumption in individual attitudes and choices rather than in systemic conditions (Shove, 2010; Spurling et al., 2013). By embedding material decisions within shared interpretative frameworks through collective intelligence knowledge creation, the adapted FLL makes anticipatory assumptions collectively visible and negotiable, shifting the analytical unit from the individual to the group. This is a methodological rather than a transformative claim: the framework does not itself overcome the individualization of climate responsibility or generate collective action, but it creates a setting in which the socially shared character of consumption norms can be examined and, following Shove (2010), in which fundamental questions about the structural conditions of sufficiency transitions can be reopened.

The aim of this adaptation is not to present decluttering as a comprehensive sustainability solution. Indeed, decluttering practices can generate rebound effects when disposal is followed by renewed acquisition. Instead, the method is conceptualized as a limited but structured operational tool that may support selected R-strategies—particularly Refuse, Reduce, and Rethink—by fostering collective re-evaluation of stored goods and by expanding dialogue within and between households about necessity, ownership, and future use. By making anticipatory assumptions explicit and subject to collective scrutiny, the approach seeks to expand the interpretative space within which household material decisions are negotiated.

Methodologically, the adapted FLL provides a framework for rendering anticipatory imagination empirically observable and analytically tractable—tacit to explicit (Miller, 2018, p.98). It enables both participants and researchers to trace how perspective shifts emerge within collective settings and how they may influence decisions about retention, redistribution, or disposal. By generating structured dialogue and analyzable data on collective sense-making processes, the approach offers a means of investigating sufficiency-oriented revaluation processes without presuming their durability or scale. Its relevance to climate mitigation lies in its capacity to examine and potentially support demand-side sufficiency mechanisms in materially affluent contexts, where overconsumption is structurally embedded and closely tied to global inequities.

No existing methodological approach systematically operationalizes anticipatory imagination as a collective, empirically observable process within household sufficiency research—leaving the anticipatory foundations of material retention practices analytically inaccessible. This article addresses this gap by developing and systematizing an adapted Futures Literacy Lab as a transferable methodological framework for rendering anticipatory assumptions observable and collectively negotiable in household decluttering contexts. The framework is designed to be relevant for researchers working on demand-side climate mitigation, circular consumption, participatory sustainability methods, and just transition research—fields in which anticipatory dimensions of everyday practice remain methodologically underdeveloped. Section 2 establishes the theoretical foundations across three bodies of literature. Section 3 introduces the Futures Literacy framework and its conceptual adaptation. Section 4 presents the facilitation protocol and the three ideal-typical workshop formats derived from iterative experimentation. Section 5 addresses theoretical implications. Section 6 addresses limitations and directions for future research.

## 2 Literature review

### 2.1 Sufficiency, R-strategies, and demand-side mitigation

Within climate mitigation debates, sufficiency has emerged as a demand-side strategy that complements efficiency and technological innovation by addressing absolute levels of consumption (Lorek and Fuchs, 2011). Rather than improving the environmental performance of products alone, sufficiency approaches emphasize reducing overall material throughput, particularly in high-income societies where consumption exceeds levels required for decent living standards. This perspective underscores that mitigation pathways depend not only on technological substitution but also on transformations in everyday practices and normative expectations regarding necessity, ownership, and wellbeing.

Household consumption plays a central role in this context. Sufficiency practices commonly involve absolute reduction, product longevity, and sharing or redistribution (Sandberg, 2021). These correspond to high-impact R-strategies such as Refuse, Reduce, and Rethink, as well as to inner-loop value preservation within circular economy frameworks (Haase et al., 2024). In materially affluent contexts, these strategies require not merely behavioral adjustment but renegotiation of what constitutes “value” and how value is preserved in objects (Bartl, 2014; Cooper and Evans, 2012; Nishijima, 2017).

The multidimensionality of value in consumer goods has been widely documented, encompassing economic value (Borg et al., 2020), functional value alongside social value (Laukkanen and Tura, 2022; Sweeney and Soutar, 2001), and emotional or epistemic value (Van Den Berge et al., 2020). Additionally, there are opinions that value does not inhere in objects but emerge through practices—through what people do with things (Arnould, 2013; p.130). Valorization is therefore a multidimensional, ongoing, socially embedded process.

Awareness of multiple value dimensions also has practical implications for sufficiency-oriented strategies. Gygi (2022) describes decluttering as a practice that reveals hidden connections between subjectivity and ethics. These connections carry inherent value systems. Making these dimensions explicit, recognizing the functional, emotional, social, and epistemic aspects of an object—expands the possibilities for revaluation, offering greater flexibility when one dimension becomes contextually impractical. Furthermore, engaging with value multiplicity may strengthen motivation—a prerequisite across all high-impact R-strategies—by deepening the perceived significance of everyday objects and the practices through which they are maintained.

While value preservation and revaluation are central to sufficiency strategies, the anticipatory dimensions through which value is projected into the future remain underexplored. Everyday life is saturated with imagined futures and the interpretative frames through which they are constructed (Miller, 2018, p.2). Decisions about retention or disposal are guided not only by present utility but also by expectations about possible future uses, identities, and contingencies which are partially situated in the realm of imagination. Although consumer research has acknowledged the role of imagination in consumption Hirschman and Holbrook (1982), linking it to desire (Belk et al., 2003) and the joys or pleasures of consumption (Campbell, 2005; Seregina, 2014) —and more recent studies suggest that imagination may also have anti-consumerist effects (Heath and Nixon, 2021)—its integration into sufficiency-oriented research remains limited.

More broadly, efforts to promote sufficiency often focus on individual consumption restraint without addressing the normative anticipatory assumptions that underpin material practices—particularly the equation of future security, identity, and social belonging with ownership and accumulation. From this perspective, sufficiency involves renegotiating socially embedded imaginaries of necessity, entitlement, and value. Such renegotiation necessarily engages anticipatory dimensions of consumption, as expectations about future needs strongly influence present retention and acquisition decisions.

Yet methodological tools for investigating and facilitating this anticipatory redefinition remain limited. Existing approaches seldom provide structured settings in which future-oriented assumptions can be collectively surfaced, questioned, and reconfigured. Against this backdrop, household material stocks can be understood as tangible sites where demand-side mitigation dynamics become visible. Decluttering practices, in particular, represent a concrete field in which sufficiency strategies may be enacted, contested, or undermined.

### 2.2 Reflective decluttering and functional revaluation

Decluttering has attracted growing attention in both popular discourse and emerging sustainability research as a potential entry point for more resource-conscious consumption. Empirical studies remain limited (Muster et al., 2022), but existing research predominantly associates decluttering with emotional and psychological benefits, such as reduced anxiety and increased wellbeing (Blackburn et al., 2023; Eike et al., 2021), or with minimalist lifestyle orientations (Matthews et al., 2015; Uggla, 2019). More critical perspectives situate decluttering within practices of domestic labor and household care (Muster et al., 2022), highlighting its embeddedness in everyday material management or as relational revaluation practice between owners and their belongings (Eimterbäumer and Jaeger-Erben, 2025).

Structured decluttering approaches rely centrally on reflection as a decision-making mechanism. Reflection functions as an evaluative process through which individuals assess possessions in relation to present utility, emotional attachment, and lifestyle compatibility. Empirical work on reflective decluttering demonstrates that such structured reflection can foster awareness of consumption habits and support strategies associated with sufficiency, including conscious purchasing, consumption tracking, and lifecycle extension (Iran et al., 2024; Muster et al., 2022). In this sense, reflection operates primarily as a monitoring and alignment task: individuals reassess belongings against existing goals, perceived needs, and established value hierarchies.

While these reflective practices may support incremental behavioral adjustments, they remain largely functionally evaluative. The focus lies on optimizing current consumption patterns rather than interrogating the anticipatory assumptions that shape expectations of future necessity, ownership, and security or value in general. Moreover, the reflective process is predominantly framed at the individual level emphasizing self-reflection during sorting (Iran et al., 2024) rather than collective negotiation of socio-material norms.

The social dimensions of decluttering have received limited attention in existing research. (Gygi 2022, p.203) goes so far as to argue that prominent decluttering approaches neglect the social entirely, characterizing the KonMari method—arguably the most widely recognized decluttering framework—as “solipsistic” in its exclusive focus on the individual's affective relationship with objects. Yet Gygi (2022) own analysis of garage sales in Japan points toward a more promising social dynamic: sellers derive satisfaction from imagining the possible future uses of their belongings in the hands of new owners, suggesting that the anticipatory imagination of objects' biographies beyond individual possession is a naturally occurring and affectively meaningful practice.

From a climate mitigation perspective, the limited attention of social dimension in decluttering is consequential. If sufficiency transitions in high-consumption societies require renegotiating what counts as “value” then decluttering must be conceptualized not merely as a behavioral sorting practice but as a site of anticipatory negotiation over socio-material norms that influence material throughput. Yet existing decluttering research has not systematically operationalized collective speculative futuring as a method for examining how such renegotiation might occur.

Taken together, reflective decluttering demonstrates potential for raising sufficiency-oriented awareness but lacks a methodological framework capable of tracing collective anticipatory revaluation processes. This gap motivates the integration of structured anticipatory approaches into decluttering research, as developed in the following section.

### 2.3 Anticipation of futures as analytical lens for consumption practices

Although attitudes and mindset are recognized as relevant for product longevity (Haase et al., 2024; Van Nes and Cramer, 2005), existing approaches rarely provide a structured framework for examining how individuals imagine the futures of their possessions and how such anticipations shape retention or disposal decisions. Anticipation studies offer a conceptual lens for addressing this limitation.

In this perspective, futures are not predetermined trajectories but socially constructed imaginaries that organize present action (Miller, 2018; Louie, 2019). Anticipation refers to the capacity to construct and model alternative futures through self-reflective processes. As Mangnus et al. (2021), drawing on Andersson (2018), Beckert (2016), and Appadurai (2013), emphasize, imagined futures inform meaning, stimulate action, and guide present choices. In (Miller 2018, p. 7) terms, the future functions as an anticipatory system: a set of assumptions and processes through which actors interpret and respond to emerging possibilities.

From this perspective, consumer practices are deeply anticipatory. Decisions about acquiring, retaining, or discarding objects are guided by implicit projections of future utility, identity, security, and social belonging. And although the future utility is assessed in various studies (Eimterbäumer and Jaeger-Erben, 2025; Muster et al., 2022; Iran et al., 2024) the implicit assumptions of the imagined futures are not examined. Household accumulation can therefore be understood not merely as a response to current and projected needs but as a materialization of anticipated contingencies. Stored goods often embody imagined scenarios—possible reuse, hypothetical life events, projected role changes—through which actors seek to stabilize uncertainty.

Anticipatory practices are not normatively neutral. Imagining the future inevitably involves assumptions about what is desirable, necessary, or legitimate (Mangnus et al., 2021). In affluent consumption contexts, such anticipatory assumptions frequently equate preparedness and wellbeing with continued possession. Rendering these assumptions explicit is analytically significant, as it reveals the value orientations that stabilize high-consumption norms and material retention practices.

At the same time, anticipation contains the potential for perception change. Because imagined futures are constructed rather than given, they can be surfaced, compared, and critically examined. Distinguishing between probable futures (based on extrapolated trajectories) and preferred futures (based on normative aspirations) (Miller, 2018) creates analytical space for questioning established links between security and ownership or between identity and consumption. In the context of sufficiency transitions, such interrogation of anticipatory assumptions is particularly relevant, as expectations about future needs strongly influence present material throughput.

Despite this theoretical richness, anticipation has rarely been systematically integrated into consumption research as a structured analytical focus. The anticipatory dimensions through which socio-material norms are stabilized or destabilized therefore remain underexplored within studies of household material practices. Addressing this gap requires methodological approaches capable of eliciting and examining anticipatory assumptions in relation to everyday material arrangements. In the following paragraph the methodological framework of Futures Literacy called Futures Literacy Lab will be introduced.

## 3 Futures literacy as research tool

### 3.1 Anticipatory assumptions and futures

Futures Literacy (FL) is a human capability concerned with the conscious and purposeful use of imagination to introduce the future into the present. As (Miller 2018, p.16) defines it, a futures literate person has acquired “the skills needed to decide why and how to use their imagination to introduce the non-existent future into the present.” This capacity is not about predicting what will happen but about becoming aware of the anticipatory assumptions — the implicit premises and frames — through which actors construct imagined futures and orient present decisions and actions. Because these assumptions typically operate tacitly, most people deploy anticipatory systems without conscious awareness of doing so, and therefore rarely consider that the future can be used for different reasons and with different methods (Miller, 2018, p.17).

Futures Literacy conceptualizes anticipation as a fundamental dimension of social practice, emphasizing how imagined futures shape present action (UNESCO, 2026; Miller, 2018). Central to this perspective is the notion of anticipatory assumptions (AA), defined as the underlying premises through which futures are constructed and rendered meaningful (Miller, 2018, pp. 4–5). These assumptions constitute the basic units of anticipatory systems (Rosén, 1985), structuring how actors interpret uncertainty and orient present decisions.

(Miller 2018, p.59) distinguishes between two fundamental modes of human anticipation: anticipation-for-the-future (AfF) and anticipation-for-emergence (AfE). AfF refers to goal-oriented anticipation in which imagined futures extrapolate from present trajectories. (Miller 2018, p.59) identifies two forms of AfF: optimization, in which futures are projected forward from current trends — as in trend extrapolation or forecasting — and contingency, which addresses uncertainty through structured methods such as Delphi techniques and simulation models. Both forms share a common logic: the future is treated as a destination to be reached, managed, or prepared for, and present action is oriented accordingly. In consumption contexts, AfF typically underpins retention practices justified through projected future utility, precautionary reasoning, or identity continuity — the implicit assumption being that holding onto objects secures a future that is essentially an extension of the present.

AfE, by contrast, treats the future as a source of novelty rather than a predetermined outcome. Rather than extrapolating from existing trajectories, AfE suspends dominant assumptions and opens space for alternative configurations of meaning and practice to emerge (Miller, 2018, p.59). This is the mode of anticipation that Futures Literacy specifically cultivates: whereas AfF-based methods such as Delphi, scenario simulation, and trend extrapolation are well suited to mapping probable futures within existing frameworks, they are not designed to surface or reconfigure the anticipatory assumptions that generate those frameworks in the first place. AfE does precisely this — it creates the conditions under which taken-for-granted frames, including those linking security, identity, and wellbeing to material possession, can become visible as contingent constructions rather than necessary constraints. In the context of household decluttering and sufficiency-oriented climate mitigation, this distinction is analytically consequential: it provides the conceptual basis for identifying when participants are operating within existing interpretative frames and when they are beginning to move beyond them, which is the core mechanism the adapted FLL is designed to activate and examine.

In this study, anticipatory assumptions are treated as the primary analytical object. Household accumulation and decluttering decisions are understood as material expressions of AfF-oriented assumptions regarding value, security, and necessity. The AfF/AfE distinction provides a conceptual lens for examining how such assumptions stabilize or potentially reconfigure socio-material practices relevant to sufficiency transitions.

### 3.2 Collective intelligence knowledge creation

While anticipatory assumptions structure present action, they typically operate tacitly. Routine “uses of the future” are rarely recognized as contingent constructions (Miller, 2018). Drawing on Dewey (1997) understanding of inquiry, Futures Literacy frames learning as beginning with interruption—disrupting habitual patterns in order to generate awareness of underlying assumptions.

(Miller 2018, p. 98) argues that perceiving anticipatory assumptions requires suspending automatic projections of the future and provoking recognition that imagined scenarios are neither inevitable nor neutral. This process, rooted in methodologies of action learning, does not aim at prediction but at exposing how expectations are constructed and how they influence present decisions.

Crucially, such learning is conceptualized as collective rather than purely individual. Anticipatory assumptions are socially embedded and reinforced through shared narratives and cultural norms (Mangnus et al., 2021). CIKC refers to structured processes in which participants collaboratively articulate, compare, and examine their anticipatory assumptions of the future that enable people to sense and make sense of present (Miller, 2018). Through dialogue, assumptions that appear self-evident at the individual and collective level can become visible as contingent and negotiable. As (Miller 2018, p. 108) notes: “...the value of CIKC lies in its emphasis on diversity and the negotiation of shared sense-making.”

Building on this, (Miller 2018, p.107) argues that adopting a relational ontology reveals a deeper potential for CIKC: rather than functioning as a bridge-building exercise between distinct and bounded actors, CIKC constitutes a generative process through which new collective understanding emerges from the dynamic interaction of parts and wholes. From this perspective, knowledge is not aggregated from pre-existing individual positions but produced through the relational connections that form between participants, their material contexts, and their imagined futures. This relational dimension is not unique to Futures Literacy research. Within circular economy scholarship, Jaeger-Erben et al. (2025) have operationalized an analogous relational logic through the Meta-Principles of Relate, Responsibilize, and Resonate—a framework that deliberately moves beyond individualistic approaches to R-strategies by embedding circularity in the relational dynamics between people, objects, and social contexts. The convergence of two distinct research traditions — anticipatory systems theory and circular economy practice — on the necessity of relational approaches suggests that the collective intelligence knowledge creation process central to the FLL design is not a methodological preference but a substantive theoretical requirement for investigating sufficiency-oriented revaluation processes at the collective rather than individual level.

Within the present framework, collective interruption serves an analytical function: it renders anticipatory assumptions empirically observable and opens space for examining how they shape socio-material valuations. It enables systematic investigation of the anticipatory foundations of household material practices.

### 3.3 Socio-material revaluation through perception shift

When anticipatory assumptions are collectively surfaced and AfE-oriented imagination is engaged, perception shifts become possible (Miller, 2018, p.23). Whereas AfF stabilizes existing interpretations of value and necessity, AfE creates distance from habitual projections and enables alternative interpretations to be considered (Miller, 2018, p.23).

In the context of household clutter, such perspective shifts may alter how objects are situated within socio-material networks. Revaluation refers here to changes in the interpretative framework through which objects are understood, whether in terms of utility, emotional significance, ownership, or redistribution. A particular benefit of implementing perceptions change through anticipating futures is that imagining futures are inherently normative and implicitly political (Mangnus et al., 2021), thus expanding the interpretative space may also surface broader considerations related to socio-economic and ecological justice. By suspending habitual anticipatory logic, household engaging with FL may become more aware of how their material practices are embedded within wider systems of production, extraction, and inequality. Revaluation does not presuppose specific behavioral outcomes; rather, it concerns the reconfiguration of meaning that precedes decisions about retention, sharing, or disposal.

This framework positions structured AfE engagement as a conditional mechanism through which the interpretative foundations of household material practices can be examined within sufficiency transitions.

### 3.4 The contingent pathway from revaluation to sufficiency

Revaluation does not lead to sufficiency automatically. Expanding the meanings and uses attributed to a stored object may support sufficiency, but it may equally furnish new reasons to retain an unused object or stimulate acquisition of similar goods. The relationship is therefore contingent, and its climate relevance depends on two dimensions: the kind of relationship a person holds with an object, and the circularity practices that relationship can support.

The first dimension concerns the person–object relationship. Actively using and caring for objects is the most effective way to preserve their value (Ellen MacArthur Foundation, 2015), keeping them in what (Eimterbäumer and Jaeger-Erben 2025) term dynamic storage. Static storage arises from passive relationships — the object forgotten or never used — or relationships in limbo, where the person cannot decide whether to keep or discard it (Eimterbäumer and Jaeger-Erben, 2025). Clutter is precisely the object held in passive or limbo relationships: dormant, unexamined, disconnected from use. Revaluation's primary contribution is to re-establish an active relationship — the interpretive effort (Eimterbäumer and Jaeger-Erben 2026) call consumption work — which is the precondition for any subsequent sufficiency-relevant practice.

The second dimension concerns what an active relationship enables: continued and attentive use, repair, repurposing, gifting, redistribution, sharing, or exchange. None is guaranteed by revaluation alone, and each relates differently to circularity. Crucially, retention is not itself a failure of sufficiency. An object retained within an active relationship remains in dynamic storage and continues to displace demand for new production; retention becomes a problem only when the object returns to static storage. The relevant distinction is thus not retention vs. disposal, but active vs. static relationships.

Conducted collectively, revaluation may also extend toward shared responses — for instance, neighborhood organizing around clutter or a specific category such as electronics. Even absent immediate material practice, the collective surfacing of awareness, such as of overconsumption or planned obsolescence, is a meaningful precondition for sufficiency-oriented action, though not sufficiency itself. Consistent with §4.3 and §5.1, this is understood as an in-session, consciousness-level outcome that may enable later practice, not as evidence of durable change.

Revaluation therefore contributes to sufficiency when it moves objects out of static storage into active relationships expressed through circularity practices, and works against it when it returns objects to static storage or stimulates acquisition. The adapted FLL is best understood not as a technique that produces sufficiency, but as a setting in which the anticipatory assumptions governing the active–static relationship can be surfaced, examined, and reconfigured.

### 3.5 Futures literacy lab setting and its adjustment for decluttering workshops

The Futures Literacy Lab (FLL) is designed as a general-purpose methodological framework adaptable to diverse research contexts (Miller, 2018, p. 39). While its core structure remains constant, thematic focus, prompts, and facilitation techniques are adjusted according to the research hypothesis. In this study, the FLL is adapted to investigate anticipatory assumptions related to the perceived value of household clutter and to examine processes of socio-material revaluation.

The standard FLL design consists of three sequential phases—Reveal, Reframe, and Rethink—with an optional Act phase. These phases are preserved but operationalized specifically to elicit and analyze the effect of anticipatory assumptions concerning the perceived value of stored consumer goods.

*Phase 1: Reveal — Surfacing Anticipatory Assumptions*

The Reveal phase aims to externalize existing anticipatory assumptions (AA) by inviting participants to articulate expected and desirable futures related to a chosen topic. The temporal horizon is selected such that precise prediction becomes implausible, encouraging imaginative projection beyond immediate planning (Miller, 2018, p. 103).

In the decluttering adaptation, participants describe future scenarios that justify retaining specific objects. Methods may include storytelling, visual mapping, collage, or structured approaches such as causal layered analysis, depending on facilitation context. This phase primarily captures anticipation-for-the-future (AfF) logics, revealing how retention practices are stabilized through projected utility, precautionary reasoning, or identity continuity.

The analytical focus is the identification and documentation of anticipatory assumptions expressed in relation to specific objects.

*Phase 2: Reframe — Enabling Anticipation-for-Emergence*

The Reframe phase introduces deliberate interruption by modifying or suspending assumptions surfaced in Phase 1. Participants are encouraged to construct alternative futures through playful and speculative engagement, enabling anticipation-for-emergence (AfE).

Rather than extrapolating present trajectories, participants explore futures in which dominant ownership norms, need structures, or value hierarchies are altered. The objective is not to prescribe sufficiency-oriented outcomes but to expand the interpretation space within which stored objects are understood.

The analytical focus lies in tracing shifts in narrative framing, justificatory language, and articulated meanings attached to objects.

*Phase 3: Rethink — Consolidating Insights*

The Rethink phase consolidates insights generated during reframing by reflecting on the multiple futures constructed and what they reveal about the present. Participants engage in collective reflection guided by questions such as: What can be learned from these imagined futures about current assumptions and practices? What forms of novelty emerged? What new questions arise concerning the present and the stored consumer goods? Which previously overlooked aspects of these goods become visible?

The analytical focus lies in identifying potential perceptions shifts regarding the stored consumer goods and instances of socio-material revaluation.

## 4 Workshop outline the “Futures of Clutter”

This paper develops a conceptual-methodological framework. The framework was not derived from a systematic empirical study but emerged from, and was refined through, a small number of illustrative developmental workshops. These workshops are best understood as developmental experiences rather than as a dataset: they provided the practical basis from which the three ideal-typical formats were shaped and through which the framework's facilitation logic was tested and adjusted. Accordingly, the examples presented in the following sections are offered to illustrate how the framework operates in practice, not as empirical evidence of its outcomes or effectiveness. The claims made about what the adapted FLL can render observable are specified further in §4.3.

The framework was developed through three illustrative workshops conducted across two settings. The first was held at Brandenburg University of Technology (BTU) Cottbus-Senftenberg with 23 international undergraduate students enrolled in an introductory course on transdisciplinary research within the Department of Environmental Sociology and Technology; participants were recruited through the course. The second and third were held with an urban gardening initiative in Karlsruhe, Germany, with five and six participants respectively, recruited through the initiative's social media and newsletter. Each workshop ran for a maximum of approximately 4 h. Across the three settings, different configurations of the Futures Literacy Lab were tested in relation to clutter and storage spaces. Following each workshop, the facilitation team reflected on the experience, and these reflections informed adjustments to the subsequent workshop and the progressive shaping of the three ideal-typical formats. For a clearer overview, several figures and tables are provided. Figure 1 illustrates how workshop procedures, the FLL logic, and revaluation processes are intertwined. Table 1 presents an exemplary workshop outline with a minimum duration of three hours, adapted from a workshop format developed by the University of Social Design (Hochschule für Gesellschaftsgestaltung). Figure 2 depicts the differing revaluation mechanisms of the three ideal-typical FLL formats, while Table 2 compares them along several dimensions.

**Figure 1:**
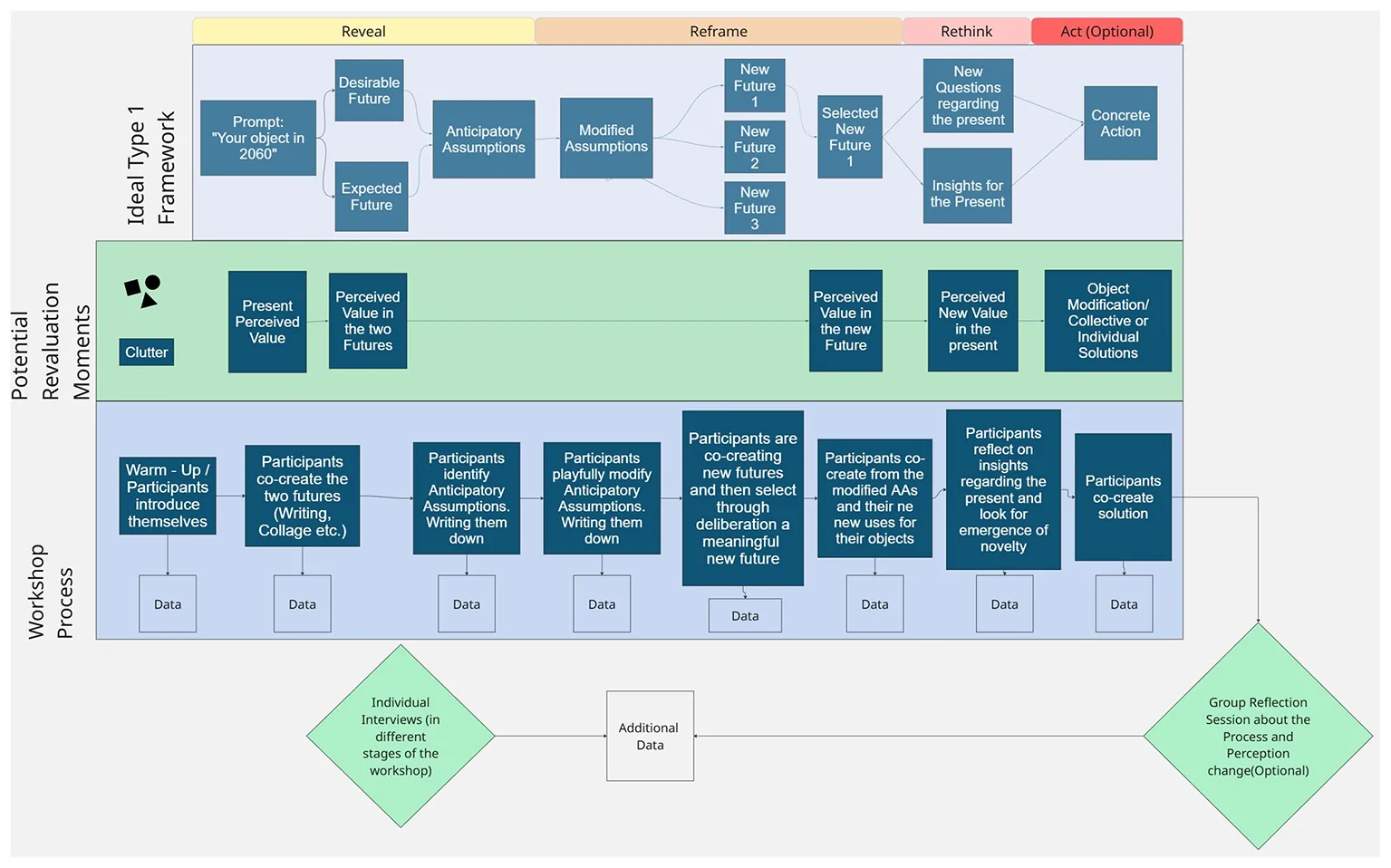
The figure maps the workshop across the four FLL phases (Reveal, Reframe, Rethink, and the optional Act phase, top) against three horizontal layers. The upper layer (FLL Process) traces the conceptual logic: an opening prompt — “Your neighborhood in 2060” (Ideal Type 1) or “Your object in 2060” (Ideal Type 2) — elicits desirable and expected futures from which anticipatory assumptions (AA) are surfaced; these assumptions are then modified to generate multiple alternative futures, one of which is selected and developed into new questions, insights for the present, and, in the Act phase, concrete action. The middle layer (Revaluation Moments) marks the points at which the perceived value of stored objects is articulated and shifts — from the object's present perceived value, through its value in imagined futures, to its value reconsidered in the present. The lower layer (Workshop Process) specifies the corresponding participant activities and the data generated at each step. Individual interviews and an optional closing group reflection session (bottom) provide additional data on process and perception change. Together the layers show how the workshop procedure operationalizes the FLL logic to render anticipatory assumptions and socio-material revaluation observable.

**Table 1:** Facilitation protocol for the adapted futures literacy lab, the “Futures of Clutter” workshop, derived from prototype workshop practice (Twiesselmann-Steigerwald et al., 2024).

Phase	Step	Method	Organization	Data collected
**Warm-up**	Check-in and expectation sharing	Participants briefly introduce themselves and state their expectations for the session.	Plenary	Facilitator notes
	Object introduction	Participants introduce their selected object using a structured storytelling format (“story of me,” “story of you,” “story of all of us”).	Plenary or small groups	Written narratives, retained as an in-session reference point
**Reveal**	Surfacing anticipatory assumptions	Participants respond to the prompt “What futures do you imagine for this object?”, documenting assumptions about value, utility, and projected future uses.	Individual written work	Written assumption statements
	Activating imagination	A brief imaginative exercise invites participants to project themselves into the chosen future year (e.g., 2060) to loosen habitual anticipatory frames.	Individual or plenary	Facilitator notes
	Sharing assumptions	Participants share their documented assumptions with the group.	Plenary discussion	Facilitator notes on group dynamics and recurring assumption patterns
**Reframe**	Assumption-flipping	The facilitator returns participants' own documented assumptions to them as contingent constructions; participants explicitly invert or suspend each assumption.	Small-group work	Written flipped assumptions
	Alternative-future construction	From the flipped assumptions, participants collaboratively construct and narrate an alternative future for their object, using storytelling, visual mapping, or collage depending on facilitation context.	Small-group work	Written or visual alternative-future narratives
	Presenting alternative futures	Groups present their alternative futures to the plenary and discuss what the reframing revealed.	Plenary	Facilitator notes on reframing dynamics and assumption resistance
**Rethink**	Structured reflection	Participants respond in writing to guiding questions: What has become newly visible? What has shifted in your understanding of the object? What do these imagined futures reveal about current assumptions and practices?	Individual written work	Written reflections, analyzed against the warm-up reference point
	Collective sense-making	Participants share reflections and identify shared or divergent insights across the group.	Plenary discussion	Facilitator notes on collective in-session reframing
**Act (optional)**	Strategy formulation	Where feasible, participants formulate individual or collective strategies for addressing stored consumer goods at household, neighborhood, or community level.	Small-group or individual work	Written or oral strategy statements
	Material experimentation	Where resources allow, participants engage directly with objects through repair, modification, or disassembly for alternative uses.	Hands-on workshop activity	Facilitator notes and participant-produced material outcomes
**Closing**	Group reflection	Participants collectively articulate what has changed in their understanding across the session.	Plenary circle	Facilitator notes or written participant responses
	Written evaluation	Participants complete a brief written evaluation of the session.	Individual written work	Evaluation forms, retained for trustworthiness assessment

The protocol is a flexible guide rather than a fixed script: facilitation methods within each phase may be adjusted according to participant composition, available time, and research objectives, provided that the core phase logic and data-collection procedures are maintained. Minimum total duration approximately 3 h. The Act phase is optional and recommended where the research context is explicitly transformative (see Section 4.3). Data-collection outputs are calibrated to what a single workshop can make observable; they document in-session reframing rather than durable perception change (see Sections 4.4 and 5).

**Figure 2:**
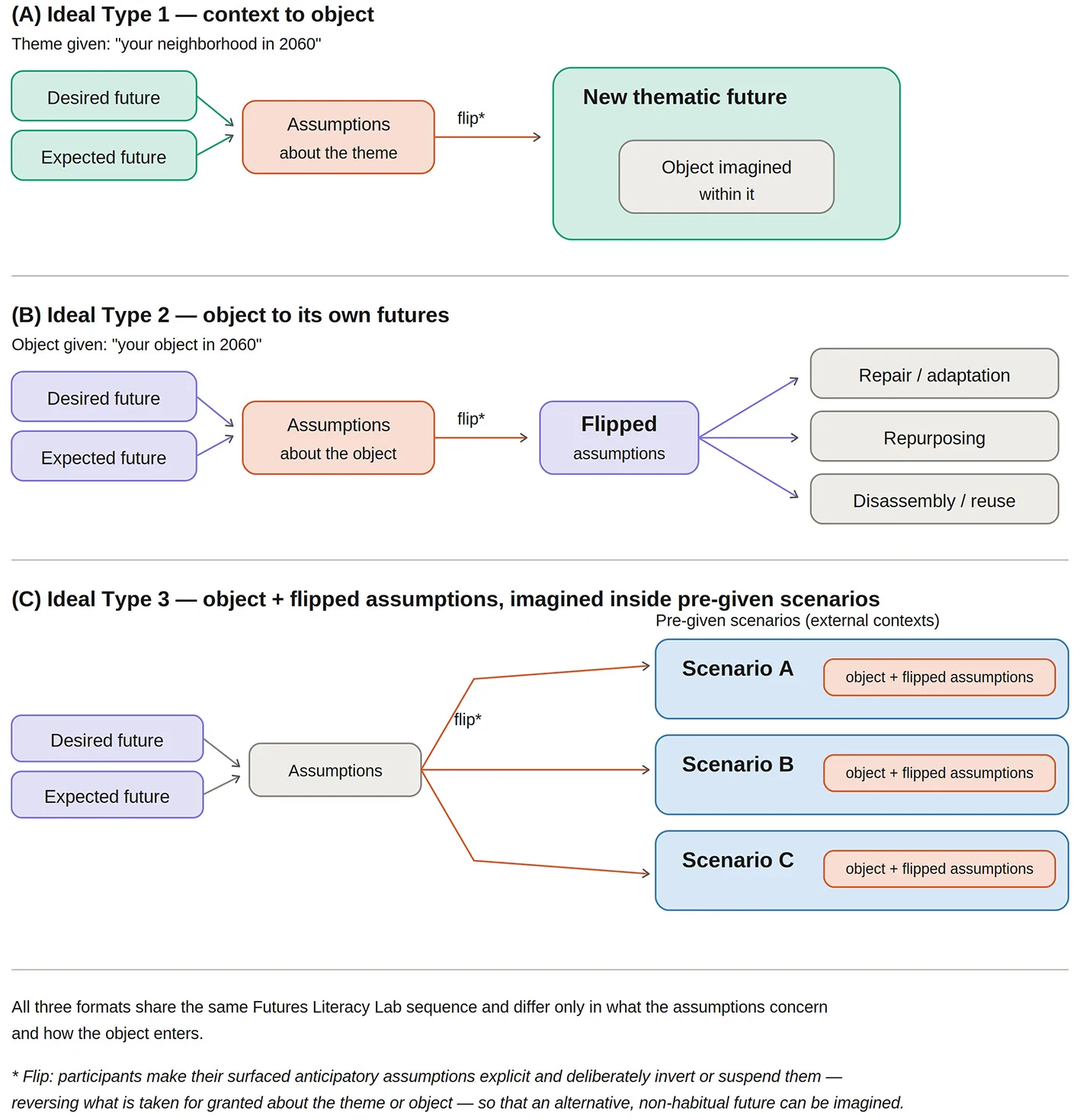
Mechanisms of revaluation across the three ideal-typical workshop formats. Each panel shows how a stored object is revalued and how anticipatory assumptions are engaged, with the direction of revaluation differing across the three formats. **(A)** In Ideal Type 1 (contextual thematic futures), an imagined thematic future is constructed first and the object is placed within it, so that the altered context reframes the object's value (context → object). **(B)** In Ideal Type 2 (object-centered projection), the object is the starting point: its anticipatory assumptions are surfaced and loosened, and future trajectories such as repair, repurposing, and disassembly are drawn out of the object itself (object → its own futures). **(C)** In Ideal Type 3 (scenario-based reframing), participants flip the object's anticipatory assumptions and imagine the object, so altered, inside a set of externally provided scenarios; the scenarios are pre-given contexts for comparison rather than products of the flip (object + flipped assumptions → pre-given scenarios).

**Table 2:** The three ideal-typical workshop formats compared.

Dimension	Ideal type 1: contextual thematic futures	Ideal type 2: object-centered projection	Ideal type 3: scenario-based reframing
**Prompt**	“Your neighborhood in 2060”	“Your object in 2060”	Flip the object's assumptions, then imagine it within pre-given scenarios
**Starting point**	An imagined thematic future	The object itself	The object and its anticipatory assumptions
**Role of assumptions**	Shift through the imagined future context	Surfaced and loosened, then projected beyond	Explicitly flipped by participants
**Direction**	Context → object	Object → its own futures	Object + flipped assumptions → pre-given scenarios
**Revaluation mechanism**	The object is placed inside a new thematic future, which reframes its value	Future trajectories are drawn out of the object by projecting it beyond its assumptions	The object, carrying its flipped assumptions, is imagined inside externally provided scenarios
**Framing**	Open, participant-generated	Open, participant-generated	Constrained, externally provided
**Primary strength**	Systemic and socio-ecological awareness	Material and functional imagination	Controlled comparison across futures
**Main watch-out**	Object may recede from view	Less engagement with systemic context	Scenarios may over-constrain openness
**Best suited when the interest is**	Situating objects in broader futures	Object trajectories and material practices	How altered assumptions play out across macro-trend contexts

Each format differs in its starting point and in the direction of revaluation: from context to object (Ideal Type 1), from object to its own futures (Ideal Type 2), and from the object's flipped assumptions into pre-given scenarios (Ideal Type 3). The three formats are shown as complementary rather than hierarchical; format choice depends on the research question, facilitation context, and participant composition.

### 4.1 Prototypical modes of embedding objects in FLL

The core adaptation of the conventional FLL lies in combining object-centered facilitation with a broadened thematic framing and, where appropriate, the inclusion of an Act phase that invites exploration of collective solution pathways. Rather than representing isolated case findings, the ideal types synthesize these iterative methodological insights into a transferable workshop design.

Ideal Type 1: Contextual Reframing through Thematic Futures (Figure 1)

In the first format, participants are invited to imagine a broad thematic future — such as “the future of your neighborhood in 2060.” Stored objects are not directly projected into the future; rather, they are reconsidered within the imagined transformed context. Clutter is thus interpreted against the anticipated shifting socio-material conditions of a particular setting in that future year — a neighborhood, an urban garden, a school, or a city — including contingent conditions imagined by participants such as resource scarcity, socio-ecological urban transformation, or reconfigured technological infrastructures. An illustrative example from the developmental workshops is the reinterpretation of a scalp massager, initially regarded as forgotten clutter, whose metal tips were reimagined as a seed-planting tool within a scenario of post-scarcity urban gardening imagined by the participants. Revaluation in this format occurs indirectly: objects acquire new meaning through their potential roles within altered but contextually specific future environments. This approach is particularly effective in situating individual possessions within broader structural and socio-ecological contexts, expanding the interpretative horizon from private ownership toward collective and systemic dimensions.

Ideal Type 2: Object-Centered Future Projection (Figure 1).

In the second format, participants are directly invited to imagine the future of a specific object in 2060. The focus lies on the potential trajectories, transformations, or reuses of the object itself. Revaluation emerges here through temporal extension of the object's biography: participants explore repair, adaptation, disassembly, or reinterpretation of material properties, progressively situating the object within alternative contexts and relational networks. This format tends to foreground functional and material aspects more strongly than systemic ones. While it can enable concrete reinterpretations and practical imagination, it may generate less engagement with broader socio-political contexts unless explicitly facilitated through additional prompts connecting object trajectories to structural conditions.

Operationally, the workshop prompt may take the form of “Your object in 2060.” Participants need not project the exact physical object they brought to the workshop; rather, the exercise may address the broader material category to which the object belongs — such as a bicycle helmet, a paper clip, or an audio device. This design choice allows participants to move beyond the contingencies of a single artifact and explore more generalizable trajectories of material practices. An illustrative example from the developmental workshops is a tennis ball, initially considered unsuitable for play, which was first reinterpreted as a massage tool and subsequently imagined as a reusable door buffer following disassembly — demonstrating how object-centered future projection can progressively expand the interpretative horizon of a single material item across multiple use trajectories.

Ideal Type 3: Scenario-Based Reframing through Pre-Constructed Futures (Figure 2).

The third format introduces pre-developed future scenarios reflecting socio-technical or socio-ecological trajectories — such as techno-optimism, socio-ecological transformation, or post-growth urbanism. Participants situate their objects within these externally provided contexts, reinterpreting their value and potential uses against the normative and material conditions each scenario presupposes. This format allows for more controlled variation of anticipatory assumptions and enables systematic comparison of object valuation across different normative and political futures. It is particularly suitable for examining how specific macro-trends influence the interpretative frameworks through which stored objects are understood. However, it may constrain imaginative openness if scenarios are perceived as overly prescriptive, and facilitation should therefore preserve sufficient space for participants to negotiate and contest the scenario premises rather than simply accepting them as given.

An illustrative example drawn from the developmental workshops is the reframing of an outdated MP3 player within a scenario premised on technological authoritarianism and resource scarcity: a device initially perceived as technologically obsolete was reinterpreted as energy-resilient and practically valuable precisely because its simplicity rendered it independent of surveilled digital infrastructures. This example is particularly indicative of the analytical potential of Ideal Type 3 — the revaluation did not emerge from the object's intrinsic properties or its projected biography but was generated by the external shift in context introduced through the macro-trend scenario, demonstrating how pre-constructed futures can unlock interpretative possibilities that object-centered or thematic formats may not reach.

All three ideal types involve trade-offs between imaginative openness, object specificity, and systemic contextualization. None is normatively superior; rather, each requires adjustment depending on research objectives, facilitation context, and participant composition. Together they represent a methodological spectrum ranging from context-driven revaluation, in which objects are reconsidered against broad thematic futures, through object-driven revaluation, in which the object's own biography is temporally extended, to scenario-controlled reframing, in which externally provided macro-trend contexts generate reinterpretation through structural contrast. The choice of format should therefore be guided by the research question: whether the primary analytical interest lies in systemic awareness, material imagination, or the comparative effect of normative future contexts on object valuation.

Independent of the specific ideal type, narrative practices proved analytically significant. At the outset of the workshop, participants were invited to introduce themselves and their selected object using structured storytelling formats (e.g., “story of me,” “story of you,” “story of all of us”). These narratives function both as warm-up exercises and as an initial articulation against which in-session revaluation can be traced. A comparison between the initial object narratives and the reflections articulated at the conclusion of the workshop may offer a traceable indicator of in-session perception changes and processes of revaluation. Whether it corresponds to durable change would require longitudinal evidence (§6.2). Storytelling may be conducted orally, in written form, or through more inclusive collage-based approaches (De Rijke, 2023), depending on facilitation context and accessibility considerations.

The FLL can also be implemented iteratively or combined with complementary participatory methods, such as design thinking, role-play, or visual art exercises, to enhance material or experiential engagement. While complementary methods may vary, the core structure of the FLL must be maintained to ensure that futures and anticipatory assumptions remain central to the process. Within this structure, methodological flexibility allows experimentation with narrative and material techniques that enhance the visibility of anticipatory revaluation processes.

### 4.2 Integration of an act phase

The standard FLL design treats the Act phase as optional, recognizing that not all perspective shifts translate into immediate action and that imaginative exploration retains intrinsic analytical value independent of behavioral outcomes (Miller, 2018, p.107). In the adapted framework presented here, the Act phase is recommended — though not required — when the research context is explicitly aiming for collective solutions and when participants have sufficient time, material access, and facilitation support to move from interpretative reframing toward practical experimentation. Under these conditions, the Act phase serves a dual function: it provides participants with a structured pathway from a shared intention to actionable response, and it provides the researcher with observable indicators of whether revaluation processes extend beyond the interpretative level toward behavioral intention.

The inclusion of this phase in the present adaptation is informed by practical experience gained through Futures Literacy workshops conducted in collaboration with students of Hochschule für Gesellschaftsgestaltung Koblenz (University for Social Design) (Twiesselmann-Steigerwald et al., 2024), as well as independent observations from workshop applications conducted with business practitioners and activists at the same institution. Across these settings, a recurring pattern emerged: participants frequently expressed a desire for concrete takeaways at the close of the workshop — including practical ideas for organizing collective initiatives around the topics surfaced during reframing. This observation proved analytically significant: the appetite for actionable outcomes functioned as a motivational resource, suggesting that the Act phase not only serves as a research function but also operates as a moment of closure and empowerment that may sustain participant engagement with sufficiency-oriented practices beyond the workshop itself.

Where feasible, participants may formulate individual or collective strategies for addressing stored consumer goods at household, neighborhood, or community levels. Depending on the workshop setting, available resources, participant skills, and facilitation goals, the Act phase may also include direct material engagement with objects — ranging from repair and functional upgrading to aesthetic modification or disassembly for alternative uses. Introducing this dimension of physical manipulation extends revaluation beyond discursive reframing and enables socio-material experimentation, making the transition from anticipatory imagination to material action observable within the session.

### 4.3 Recommended data acquisition protocol

Systematic data acquisition across the FLL phases serves a dual purpose: it provides the researcher with traceable material for analyzing anticipatory assumptions and revaluation processes, and it creates a within-session reference point against which in-session revaluation can be documented. The aim of Futures Literacy, and therefore of the FLL, is a change in perception achieved by using the future to reinterpret the present (Miller and Sandford, 2019). In the adapted clutter FLL, however, this aim must be carefully qualified in terms of what a single workshop can make observable. It is therefore important to distinguish clearly among the different levels of output the adapted FLL can render observable, since these carry different evidential weight. The workshop directly makes observable: (1) the anticipatory assumptions elicited during the workshop; (2) the alternative framings/futures generated through the reframing exercise; and (3) participants' reported insights at the point of reflection. It does not, on its own, establish (4) durable perception shifts or changes in subsequent material practice or behavior.

Within a single workshop, no durable behavioral or sustainability-oriented perception shift can be documented; establishing these would require longitudinal designs with follow-up interviews (§6.2). What a single session can document are first insights, emergent and novel perspectives, and — where an Act phase is included — shared intentions toward collective solutions articulated within the session. These correspond to Levels 1–3 of the distinction above; they do not, in themselves, evidence Level 4. Accordingly, the comparison of narratives across a session documents in-session reframing, not a durable change of the kind that would evidence Futures Literacy as an acquired capability. The following protocol draws on data collection practices developed and tested across the developmental workshops and is offered as a methodological recommendation for future implementations of the adapted FLL.

#### 4.3.1 Warm-up phase

The primary data source in the warm-up phase is participant-produced written text generated during the storytelling exercise. Participants introduce themselves and their selected object through a structured narrative format, producing a written account that captures their initial relation to the object and, indirectly, its ascribed value, utility, and projected future. This material functions as a within-session reference point: by preserving the participant's initial framing in written form before any reframing has occurred, the storytelling output enables the documentation of in-session reframing across the workshop. Facilitators should collect all written narratives at the close of this phase and retain them as primary analytical material.

#### 4.3.2 Reveal phase

The Reveal phase generates the core analytical data of the workshop: participant-produced written articulations of the anticipatory assumptions guiding retention, disposal, and acquisition decisions — assumptions regarding the object's value, utility, projected future uses, and emotional significance. Prior to articulating their assumptions explicitly, participants first generate written descriptions of an expected and a desirable future for the object or theme; these futures are themselves primary material, since the anticipatory assumptions are subsequently identified from what participants take for granted in imagining them. Participants document the assumptions that ordinarily operate tacitly, rendering them explicit. These articulated assumptions constitute Level 1 of the output distinction above and serve as the input material for the Reframe phase. All written outputs should be collected and retained alongside facilitator observational notes.

#### 4.3.3 Reframe phase

Two distinct data types are recommended during the Reframe phase. First, participants produce written articulations documenting the process through which the assumptions surfaced in the Reveal phase are identified, challenged, and inverted — capturing the moment of assumption-flipping as it occurs. Second, participants generate written descriptions of the alternative futures that emerge from this process (Level 2 of the output distinction). In Ideal Type 1 applications, facilitators should additionally document, through notes, the group discussion in which participants negotiate which anticipatory assumptions to select for reframing and the reasoning behind their choices — a deliberative process that is itself analytically significant as an expression of collective intelligence knowledge creation. All written outputs from this phase should be collected and retained alongside the facilitator's observational notes.

#### 4.3.4 Rethink phase

The Rethink phase generates written reflections on what the alternative futures constructed during reframing reveal about present assumptions and practices (Level 3 of the output distinction: participants' reported insights). Participants record their insights in response to structured guiding questions concerning what has become newly visible, what has shifted in their understanding of the object, and what the imagined futures suggest about current socio-material norms. This material should be analyzed in direct comparison with the warm-up storytelling output to document in-session reframing; it captures reported insight at the close of the session and does not, in itself, evidence durable change.

#### 4.3.5 Act phase

Where the Act phase is included, data acquisition shifts from individual written reflection to the documentation of co-created collective outputs. Participants produce written or visual articulations of proposed individual or collective strategies for addressing stored consumer goods — ranging from practical redistribution plans to ideas for community-level initiatives. Facilitators should document these outputs in full, as they provide observable evidence of whether in-session reframing extends toward stated behavioral intention. Whether stated intention translates into subsequent practice (Level 4) lies beyond what the session documents.

#### 4.3.6 Group reflection

A structured group reflection at the close of the workshop constitutes a final data source. Participants collectively articulate what has changed in their understanding across the session, providing an additional record of reported in-session insight that complements the written phase outputs. This reflection may be conducted orally and documented through facilitator notes, or in written form depending on time and accessibility considerations. Beyond its function as a data source, the group reflection constitutes a final opportunity for in-session learning: hearing others articulate how their perceptions shifted can prompt further reframing in participants whose own assumptions had not yet moved, making the collective reflection itself a site of potential perception change rather than merely its documentation.

#### 4.3.7 Supplementary data collection

Two supplementary methods are recommended across the full workshop process. First, brief conversational interviews conducted either between phases or immediately following the workshop allow facilitators to probe emerging insights, clarify ambiguous statements, and capture reflections that participants may not have expressed in written form during the phases themselves. Second, continuous facilitator note-taking throughout the session — documenting spontaneous discussions, unexpected reframings, moments of resistance or confusion, and group dynamics — provides contextual material that enriches the analysis of phase-specific outputs and supports the trustworthiness of the analytical process. Where group size requires it, an assistant facilitator dedicated to note-taking is recommended to ensure adequate documentation without compromising facilitation quality.

For researchers seeking to extend the analytical scope of the workshop beyond the in-session levels documented here, (Gáspár 2026) demonstrates that reflexive methods combined with psychometric instruments can be integrated into Futures Literacy research designs to assess the development of futures literacy as a capacity across participants — an approach that could be adapted to decluttering contexts and that speaks directly to the Level 4 evidence the present protocol does not capture. Additionally, Bergheim (2022) provides a comprehensive account of evaluation methods developed specifically for Futures Literacy Laboratories, offering instruments for assessing participant learning, competence development, and workshop effectiveness that are transferable to the adapted FLL design presented here.

### 4.4 Analytical procedures

The analysis of the developmental workshops was exploratory: collected materials — storytelling texts, documented assumptions, alternative-future narratives, Rethink reflections, and facilitator notes — were read iteratively across workshops from which the three ideal-typical formats were derived. This exploratory mode suited the framework-development aim of establishing operability and distilling transferable design configurations rather than producing confirmatory findings.

For researchers implementing the framework as a research instrument, a more systematic procedure is recommended. Because the principal in-session output of the adapted FLL is reframing made visible through narrative comparison, the core operation is a structured within-session comparison: the warm-up narrative and the Reveal-phase assumptions form the reference point against which the Reframe and Rethink outputs are compared, rendering in-session revaluation traceable. This comparison documents Levels 1–3 of the output distinction (§4.3); it does not, in itself, evidence Level 4. Four foci structure the comparison: identification of the anticipatory assumptions surfaced in Reveal; tracing of the alternative framings generated as participants move from AfF to AfE; identification of reported revaluation, where participants articulate a reconfigured interpretative framework for an object; and participant self-reflexivity. (Gáspár 2026) identifies reflexivity — participants‘ own awareness of how they use the future — as the central measure of Futures Literacy. Although the adapted clutter FLL does not aim to measure Futures Literacy as a capability, participants' self-reflexivity concerning their own shifting assumptions about the value of stored objects remains central, and the degree to which participants recognize this shift is itself an analytical object. The materials can be analyzed through qualitative content analysis, with facilitator notes and group-reflection records providing contextual triangulation. Because assumptions are collectively produced, the analysis is related to relational group dynamics as well as individual texts; cross-workshop comparison, procedural transparency, and, where feasible, multiple analysts support trustworthiness.

## 5 Discussion

### 5.1 Beyond individual imagination

A central question raised by the adapted FLL concerns the relationship between individual anticipatory imagination, shared interpretation, collective intention and practice to structural transformation. In this article, the transition from individual imagination to shared interpretation is posited as a preliminary step that may facilitate the emergence of collective intention, which, in turn, could give rise to collective practices that may support structural transformation.

The relationship between individual imagination and collective practice invoked throughout this article spans several distinct levels, which should not be conflated. It is useful to distinguish among: collective discussion, in which participants articulate and compare their assumptions; shared interpretation, in which the group arrives at common reframing; collective intention, in which participants articulate shared intentions to act; collective practice, in which groups actually enact changed material practices beyond the workshop; and structural transformation, in which systemic conditions are altered. The adapted FLL makes the first three directly observable within a session: it documents collective discussion, shared interpretation, and — where an Act phase is included — stated collective intention. It does not, on the basis of the workshop setting, establish collective practice or structural transformation; these are proposed as directions for future research (§6.2) and depend on conditions that lie beyond the method's reach. The following discussion should be read with this graduated distinction in mind.

Anticipation is understood as sociocultural embedded from the outset: the anticipatory assumptions that individuals hold about the future value, necessity, and identity-significance of their possessions are not purely personal but are shaped by shared narratives and cultural norms (Mangnus et al., 2021). This embeddedness extends even to what appears most private: following Lizardo (2017) argument, even “personal” significance is always already encultured, such that an ordinary consumer product may be understood as a cultural object carrying shared meaning beyond mere individual attachment (McDonnell, 2023; p.198). What appears in the Reveal phase as an individual's private justification for retaining an object is therefore already a culturally patterned anticipation — one that the workshop framework setting renders visible as such.

As individuals articulate and compare their anticipatory assumptions within the group, assumptions that appeared self-evident at the individual level become visible as contingent and negotiable (Miller, 2018). This is the analytical pivot of the framework: the individual's imagined future for an object, once externalized and placed alongside those of others, is reframed as one possibility among many, opening space for shared reinterpretation. The relational dimension established earlier (§3.2) is essential here — revaluation does not emerge from individual cognition alone but from the relational field generated when multiple anticipatory framings are brought into contact, compared, and renegotiated.

This relational mechanism has implications for how the framework relates to collective practice and structural change — implications that must be stated with care. By transforming individually framed consumption choices into shared socio-material concerns, the FLL creates conditions under which group-based responses — redistribution, sharing, or collective initiatives — can be articulated and collectively intended within the session, as observed in the Act phase. The re-narration of relationships with consumer objects that Gabriel and Lang (2015) identify as a counterforce to cycles of acquisition and disposal is thus reframed within the FLL as a collectively negotiated rather than individual accomplishment. Whether this collective intention translates into durable collective practice, and whether such practice contributes to structural change, cannot be established within the workshop setting. The framework's demonstrated achievement is more modest but nonetheless significant: it opens and makes analyzable the collective interpretative space within which the possibility of collective practice is first articulated.

### 5.2 Implications for futures literacy lab

The adaptation of the FLL to household decluttering contexts carries significant theoretical implications for the Futures Literacy field. Miller (2018) framework was developed primarily for governance, education, and policy contexts in which anticipatory assumptions operate at the level of institutional and societal futures. The present adaptation demonstrates that anticipatory assumptions are equally operative—and analytically tractable—at the level of the most mundane material decisions: what to keep, what to discard, and what to imagine a stored object might become. Everyday consumer life is already normatively structured through imaginative investments in objects (Althusser, 2010) and through the routines and rituals within which consumer products are embedded (Župančič, 2008). By making objects the medium through which anticipatory assumptions are surfaced and examined, the FLL operationalizes what might be termed anticipating through objects—rendering explicit the imaginative investments that ordinarily structure material life below the threshold of conscious awareness. This extension of the FLL into everyday material practice suggests that futures literacy is not a reserved capacity for strategic or collective futures-making at the macro level but is embedded in the micro-level interpretative frameworks through which people navigate their material worlds on a daily basis. Theoretically, this expands the scope of futures literacy research beyond institutional and policy contexts toward the domain of everyday consumption, opening new possibilities for investigating how anticipatory assumptions stabilize or destabilize material practices at the household level.

### 5.3 Implications for sufficiency research

The integration of Futures Literacy into sufficiency research introduces methodological and conceptual reorientation with significant theoretical consequences. Existing sufficiency approaches have focused predominantly on what people do—their consumption behaviors, disposal practices, and lifestyle choices—without systematically examining what people imagine: the anticipatory assumptions about future security, identity, and necessity that underpin and stabilize those practices. The adapted FLL shifts the analytical focus from behavior to anticipation, proposing that sufficiency transitions may require not only practical behavioral adjustment but a prior reorientation of the imaginative frameworks through which future needs are constructed. Furthermore, by introducing a relational ontological approach—in which the potential value dimensions of stored objects are expanded through collective engagement rather than individual assessment (Jaeger-Erben et al., 2025), the FLL creates conditions under which value expansion becomes possible. This is significant: according to Haase et al. (2024), expanding the perceived value dimensions of products is a necessary condition for extending their useful lifetime, which directly contributes to the implementation of nine R-strategies within circular economy frameworks. Finally, by enabling meaning-making for and with stored objects, the FLL positions clutter not as waste awaiting disposal but as a repository of latent meaning. Objects understood as bearers of meaning — as Gabriel and Lang (2015) argue — counteract the rapid cycle of disposal and acquisition that characterizes high-consumption societies, thereby supporting sufficiency-oriented material practices at the household level and beyond.

### 5.4 Implications of the combination

The most significant theoretical contribution of this adaptation lies in what the combination of the two fields produces that neither generates alone. Futures Literacy provides a structured method for surfacing and reconfiguring anticipatory assumptions but lacks a normative anchor specifying why such reconfiguration matters for sustainability. Sufficiency research provides that normative anchor — reducing material throughput in high-consumption societies — but has lacked methodological tools for examining the anticipatory dimensions of the practices it seeks to transform. Together they produce a framework in which the imaginative foundations of overconsumption become empirically observable and collectively negotiable. This convergence suggests that futures literacy and sufficiency research are not merely compatible but mutually constitutive — each providing what the other lacks — and that their integration represents a theoretically productive direction for demand-side climate mitigation research more broadly. Furthermore, the combination of Futures Literacy and sufficiency research around questions of materiality invites a relational ontological perspective that resonates with the core commitments of new materialism — a theoretical orientation that (Benson 2019, p.251) argues constitutes the most appropriate ontological framework for environmental governance in the Anthropocene, precisely because it moves beyond individualist and anthropocentric assumptions toward an understanding of human and material agencies as mutually constitutive.

## 6 Limitations and future research

### 6.1 Limitations

Several limitations of the present methodological application should be acknowledged.

First, perspective shifts observed during the workshop do not necessarily translate into sustained behavioral change. Revaluation in this study primarily occurred at the level of meaning reconfiguration. While such shifts are analytically significant, they do not guarantee practical implementation of sufficiency-oriented practices. Imaginative engagement may remain provisional or situational.

Second, certain workshop formats, particularly Ideal Type 2 (object-centered future projection)—may produce futures that are overly abstract or insufficiently grounded in material specificity. As Gygi (2022) mentions, the future potentiality of everyday objects can be unlimited, which can be counterproductive. If facilitation does not adequately contextualize or enable meaning making group processes, participants may experience confusion or disengagement. This raises the need for experienced facilitators.

Third, the developmental workshops were conducted predominantly with participants who — through their academic, professional, or activist backgrounds — were already familiar with sustainability and sufficiency discourses. This composition introduces systematic bias. Participants already disposed toward sustainability are more likely to find sufficiency-oriented futures plausible and to produce the revaluation shifts the framework is designed to elicit. Those from academic and professional backgrounds are also more accustomed to reflective and experimental formats, and may engage more readily with the speculative, open-ended exercises the method employs — a disposition that cannot be assumed among participants less familiar with such modes of working. The framework's observed capacity to generate perception shifts may therefore partly reflect the predispositions participants brought with them rather than the effect of the method itself.

This has two implications. Methodologically, the framework's effectiveness cannot be assessed independently of participant composition on the basis of these developmental workshops; claims about what the method achieves remain provisional until tested with participants who do not share these predispositions. Substantively, it raises a question central to transferability: whether anticipatory reframing can destabilize consumption norms among participants for whom sufficiency is not already a salient value, or whether the method primarily deepens reflection among the already converted. Testing the framework across socio-demographically and ideologically diverse groups is therefore a precondition for assessing its broader validity, as elaborated in the future research directions below.

Fourth, the outcomes of an FLL are highly contingents on participant composition. Variations in creativity, material knowledge, openness to speculation, and reflexive capacity significantly influence how anticipatory assumptions are articulated and reframed. As a result, comparability across workshops is limited, and careful curation of participant groups may enhance but also bias outcomes. Workshops conducted with various Focus Groups can give more structured results.

Fifth, Futures Literacy is conceptualized as a capacity that develops through repeated practice. A single workshop may not fully unfold the transformative or reflexive potential of the method. Temporal revaluation may remain ephemeral without sustained engagement.

Finally, the adapted FLL should be understood as a complementary instrument within broader sufficiency and R-strategy frameworks. It cannot substitute for structural policy measures, infrastructural change, or material design interventions on production sites. Imaginative reframing from demand sites plays a supportive rather than primary role in practical sufficiency implementation.

### 6.2 Future research

Three priorities follow from these limitations.

First, the three ideal-typical formats should be implemented and compared systematically. Future studies should apply each format to comparable participant groups and document the data outlined in the data acquisition protocol (§4.3), to assess how each format differs in the anticipatory assumptions it surfaces, and the revaluation shifts it produces. Such a comparison would clarify which format suits which research objective.

Second, longitudinal designs should examine whether revaluation shifts persist beyond the workshop. A repeated-engagement design — for example, three to four workshops with the same group over 6 months in a neighborhood context — combined with participant diaries and follow-up interviews, would show whether initial perception shifts translate into durable changes in how stored goods are retained, redistributed, or acquired. This would test whether single-workshop revaluation remains ephemeral.

Third, the framework should be tested with more diverse participants, including groups for whom sufficiency is not already a salient value. Comparative implementation across sustainability-oriented and sustainability-skeptical groups would establish whether the method's effectiveness is contingent on participant predisposition. This is a precondition for assessing transferability beyond academically informed contexts.

Further research should also explore the interaction between anticipatory imagination and material knowledge. Participants with vocational skills or technical expertise may engage differently with revaluation processes, particularly when repair, modification, or disassembly are involved. Investigating how material competence interacts with socio-material imagination could clarify under which conditions revaluation leads to practical reconfiguration.

Finally, comparative studies involving more socio-demographically diverse participant groups would be valuable in assessing the broader applicability of the method beyond academically informed sustainability contexts.

## 7 Conclusion

The primary value of this article lies in offering a transferable methodological tool for making anticipatory assumptions visible and analytically accessible within sufficiency-oriented climate mitigation research. By operationalizing the Futures Literacy Lab for household material contexts, the adapted framework renders the imaginative foundations of overconsumption empirically observable and collectively negotiable. Its contribution is not a claim that decluttering resolves overconsumption, but a structured means of investigating how anticipatory assumptions stabilize material retention and how collective reframing might destabilize them. Adaptable across different formats and populations, the framework gives researchers in demand-side mitigation, circular consumption, and just transition research a replicable instrument for examining the anticipatory dimensions of everyday material practice — a dimension that remains methodologically underdeveloped within sufficiency research.

## Data Availability

The original contributions presented in the study are included in the article/supplementary material, further inquiries can be directed to the corresponding author.
